# Translating the Medicare Annual Wellness Visit into messages and materials to improve preventive care in older adults

**DOI:** 10.3389/fmed.2026.1919767

**Published:** 2026-09-11

**Authors:** Derjung M. Tarn, Linda Zittleman, Danielle Schramm, Nina Graham, Ray Haeme, Barbara Haeme, Grant Jones, Lenata Jones, Susan Lowe, Melanie Murphy, Willie Murphy, Nettie Oliverio, Andrew Pasternak, Ting Pun, Teri Rose, Harry Scheer, Shirley Stowe, John M. Westfall

**Affiliations:** 1 Department of Family Medicine, David Geffen School of Medicine at UCLA, Los Angeles, CA, United States; 2 Department of Family Medicine, University of Colorado, Aurora, CO, United States; 3 Patient and Clinician Engagement Group, North American Primary Care Research Group (NAPCRG), Leawood, KS, United States; 4 DARTNet Institute, Aurora, CO, United States

**Keywords:** annual wellness visit, community engagement, Medicare, older adults, participatory research, prevention, primary care

## Abstract

**Background:**

To increase the uptake of preventive healthcare services in the elderly, Medicare implemented the Annual Wellness Visit (AWV). Many patients are unaware of this free annual visit so uptake of the AWV in primary care has been low. Boot Camp Translation (BCT) is an evidence-based method for translating complex medical jargon into messages and materials for patients and primary care practices.

**Methods:**

A national community advisory council of primary care clinicians and patients eligible for Medicare benefits were recruited from primary care practices across the United States. A BCT was conducted over 7 months and included a half day in-person kickoff, 5 phone calls, and 2 virtual video conferences.

**Results:**

Thirteen community patient partners, 4 clinicians, and 2 project staff participated in the Annual Wellness Visit BCT. This group gained a deep understanding of the AWV and developed messages tailored to people over 65 who are eligible for AWVs that included: prevention, staying healthy, having more time with primary care clinicians and getting to do the activities one likes for more years. Messages included that the visit is usually free and has potential financial benefit to the practice. Participants encouraged a combination of general messages that could be tailored and customized for local regions, cities, practice and community settings.

**Discussion:**

A group of patients and primary care clinicians participating in a BCT on AWVs successfully co-created an array of engaging and compelling messages and materials to promote the AWV. Future research will test the impact of these messages and materials on patient and clinician uptake of AWVs.

## Background

The Annual Wellness Visit is a yearly free-to-the-patient Medicare benefit aimed at giving healthcare providers and patients dedicated time to focus on disease and illness prevention. In the United States, Medicare is a federal health insurance program that guarantees health coverage primarily for adults aged 65 and older and for younger people with certain disabilities or end stage renal disease. Over 90% of Medicare patients are aged 65 and older (1). Prior to 2005, Medicare did not cover any yearly visits for patients. In 2005, Medicare began coverage for a one-time Initial Preventive Physical Examination for new Medicare beneficiaries. The Annual Wellness Visit (AWV) was first introduced in 2011 with the intention of serving as an annual visit with primary care providers and their team members (2). Patients who have had an AWV are more likely to have completed preventive health screenings and have stronger relationships with their providers (3–5). However, uptake of the AWV in primary care practices has been low, with fewer than half of patients receiving an AWV in 2020 (6). Utilization has varied across racial and ethnic groups, with White patients having the highest rates (26.3%) and Hispanic patients the lowest (18.3%) (4).

Multiple challenges preclude completion of AWVs. A major barrier to patients receiving AWVs is the lack of a provider's recommendation, particularly among low-income populations and racial/ethnic minorities (7–9). Providers may have suboptimal systems to identify eligible patients and track a patient's need for preventive services (10). During time-limited office visits, acute or chronic conditions often supersede preventive health discussions (11) and may keep providers from routinely offering AWVs to patients. Patients may be unaware about the existence of and coverage for these visits and may not understand the need for preventive healthcare (12, 13). For example, many people, particularly those born in other countries, believe that cancer screening is needed only when they develop symptoms (14, 15). Some patients may not believe in the efficacy of preventive care services or may fear having tests (7, 16, 17).

The majority of the interventions designed to increase AWVs have focused on increasing organizational capacity for these visits. Strategies include enlisting non-physician healthcare providers to conduct the visits, sometimes working in a team with a physician provider (18–20). Other interventions have included patient outreach regarding the AWV but did not examine patient perceptions of the messages used (21–23). Therefore, the content of patient messaging around AWVs and how or whether they resonate with patients has been largely unexplored.

Derived from an NIH funded project, and to inform a second NIH-funded project aimed at helping practices increase their rates of AWVs (National Institute On Aging of the National Institutes of Health, Award Numbers R33AG068946 and R01AG081996) (24, 25), this project used a community-engaged process called Boot Camp Translation (BCT) to create new messages and materials regarding the AWV for patients and practices. Boot Camp Translation, also known as Community Translation, is an evidence-based, community-engaged approach that brings together community members, patients, health professionals, and researchers to translate complex medical jargon into relevant and actionable messages and materials (26, 27). Boot Camp Translation (BCT) has been used widely for projects addressing a range of health topics and with a variety of community partners (28–33). BCT is usually conducted at a local regional level within a state, and has not been used on a national level. The objective of the AWV BCT was to create messages and materials relevant to potential beneficiaries aimed at increasing the uptake of their Medicare benefit. The purpose of this manuscript is to describe the BCT process and share the resulting messages created by and for people aged 65 and older who receive Medicare benefits.

## Methods

For this project, the BCT process occurred over seven months between October 2023 and May 2024. A national group of participants was recruited from the North American Primary Care Research Group (NAPCRG) Patient and Clinician Engagement (PaCE) group, which is comprised of patients, community members, and practicing primary care clinicians (34). A snowball method was used to identify additional participants to ensure a range of perspectives, experiences, and geography within the group. The resulting group consisted of 13 community-based patient partners, 2 community primary care clinicians, two facilitators (one of whom is a primary care clinician), a physician medical/content expert (primary care physician and PI on multiple funded projects related to the AWV), and a project team member. Table 1 describes the participants. All community members were eligible to receive an AWV, and all clinicians offered AWVs in their practices. Participants in Boot Camp Translation served as lay co-researchers and were not deemed study subjects (45 CFR 46, Common Rule 2018). The parent study, NIH R01AG081996, was approved by the UCLA IRB through expedited review.

**Table 1:** Annual Wellness visit Boot Camp Translation partnership group characteristics.

No.	Role	Gender	Race/Ethnicity	Background/Interest in Project	Time Zone
1	Community Partner	Male	White, non-Hispanic	Retired	Pacific
2	Community Partner	Female	Black African American	Retired. Active University of California San Francisco patient advisor	Pacific
3	Community Partner	Female	White, non-Hispanic	Fine Artist, Art Gallery Partner, cared for elderly parents, participates in AWV yearly. rural	East
4	Community Partner	Male	White, non-Hispanic	Retired Army Officer, retired management and technical consultant, have cared for elderly parents, participates in AWV yearly. Rural.	East
5	Community Partner	Male	Black African American	Retired non-profit director	Mountain
6	Community Partner	Female	Black African American	Retired schoolteacher	Mountain
7	Community Partner	Female	White	Retired social worker, patient partner since 2013, co-investigator in 2025 Has had two AWVs with local primary care	Pacific
8	Community Clinician	Female	Black African American	Advanced Practice Nurse	East
9	Community Partner	Female	Black African American	Retired. Senior weightlifting champion	East
10	Community Partner	Female	White, non-Hispanic	Retired	Pacific
11	Community Clinician	Male	White, non-Hispanic	Family Doctor	Pacific
12	Community Partner	Male	Asian American (immigrant)	Caregiver, elderly patients, patient partner in CER research	Pacific
13	Community Partner	Female	White, non-Hispanic	Former Program Manager UCSF Division of Palliative Medicine, Patient and Family Advisory Committee (PFAC) leader, Caregiver	Pacific
14	Community Partner	Male	White, non-Hispanic	Retired	Pacific
15	Community Partner	Female	White, non-Hispanic	Nurse with background in public health, chronic disease, end of life care; Medicare consumer; AWV participant	Central
16	BCT Facilitator Clinician	Male	White, non-Hispanic	Family Doctor, retired faculty	Mountain
17	BCT Facilitator	Female	White, non-Hispanic	Researcher, experienced BCT facilitator, elderly parents	Central
18	Principal Investigator Clinician	Female	Asian American	Study Principal Investigator and BCT Content Expert, Family Doctor	Pacific
19	Program Coordinator	Female	White, non-Hispanic	Program coordinator	Pacific

### BCT process, meetings, and activities

**Figure 1:**
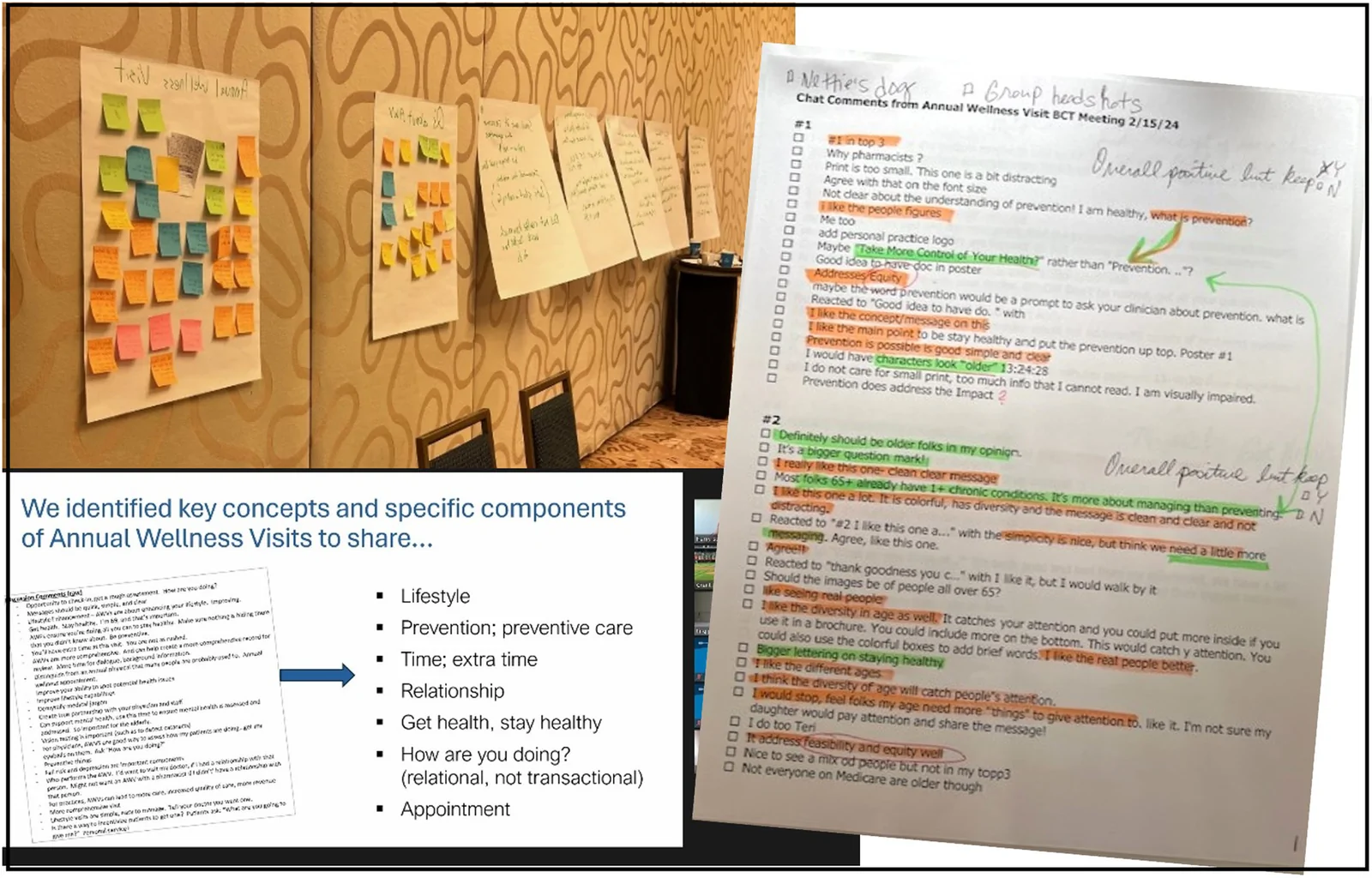
Original notes, transcribed notes, translated notes from AWV BCT.

The AWV BCT began with a half day kickoff meeting. This meeting was held in-person as part of the pre-conference activities leading up to the NAPCRG Annual Meeting, which several participants attend as part of PaCE. Per the BCT process, this first meeting included ample time for introductions and a comprehensive educational presentation on AWVs. The BCT process uses a rigorous participatory research model that attempts to level power, encourage broad participation, identify and embrace group values and communication, and assure every member has equal voice (26). Co-facilitators of the AWV BCT were members of the original team that created BCT, and attempted to fulfill the values expressed by the original community member creators. Major components of the interactive educational presentation included: history of coverage for yearly preventive visits for Medicare patients, definition of an AWV, rationale and evidence for AWVs, current utilization rates, barriers to AWV adoption, examples of questionnaires and forms that meet visit requirements, provider billing, and common provider questions. After the educational presentation, the group continued to ask questions, shared general reactions, and called out what they learned, what surprised them, and key pieces of information that they would want their friends, family, and community to know about AWVs. These topics were selected to provide a comprehensive overview of the AWV. Table 2 describes the major discussion topics identified by the patient partners during BCT kickoff meeting. The facilitators captured notes, which were saved in their raw form and then organized into “processed notes” to guide the group through subsequent stages of the BCT. Raw notes were reviewed by co-facilitators to group common themes, identify proposed message content, target audiences, and potential types of products for dissemination. Figure 1 depicts the transition from raw notes to processed notes to translated ideas.

**Table 2:** Major discussion topics identified during the BCT kickoff meeting.

Major Topic	Examples
Background	History of Medicare/Medicaid and history of coverage for yearly preventive visits and for AWVs
Why are AWVs Important?	Evidence concerning increased preventive health services use, better provider-patient relationships, increased primary care revenue, fulfillment of quality metrics, capture of hierarchical condition codes for patient risk adjustment, decreased total healthcare costs
Underutilization of AWVs	Overall rates of AWVs over time, and differences in rates of use based on patient characteristics (e.g., rural vs. urban, # comorbid conditions)
What is an AWV?	Terminology describing different types of annual visits (e.g., comprehensive physical exam, annual physical, AWV), definitions and components of different types of visits [Initial Preventive Physical Exam (IPPE), Initial AWV, Subsequent AWV], requisite timing of visits, types of providers who can perform the visits
Examples of questionnaires and forms that can be used to satisfy visit requirements	Health risk assessment, depression and alcohol screening instruments, Physician Orders for Life-Sustaining Treatment, personalized prevention plan
Billing for an AWV	How relative value units (RVUs) work, examples of screening and counseling services that providers can bill with an AWV without incurring patient co-pays or deductibles, adding modifiers for separate identifiable services
Barriers to AWV adoption	Practice, provider, and patient barriers
Common Provider Questions	How to find the time to do AWVs, how to interpret lack of requirement for a physical examination

Following the first meeting, the group held four 30-minute conference calls and two virtual meetings (2.5 and 2 h, respectively). The primary tasks were to create messages/information, determine the types of materials to use and create them, and determine distribution strategies. The first call included a review and group approval of the raw notes and the Processed Notes. These conversations tapped into partners' personal experiences, expertise, and creativity. Table 3 outlines the agenda and schedule for the 7-month process. Additional calls would usually be held between the second and third meetings in the BCT process. However, for this project, the progress made during the second meeting and information gleaned through email correspondence between meetings were sufficient for message development. Materials were created by a consulting graphic designer who worked with BCT groups for 10 years (35).

**Table 3:** Annual wellness visit Boot Camp Translation meeting and call schedule and topics.

Event	Date	Duration	Community Partner Attendance	Discussion Topics
Meeting 1 (In-person)	10/30/23	4 h	15	Overview of AWVs What they are Why needed Current utilization Barriers to using Discussion of reactions, what people learned Meeting schedule agreed upon
Call 1	11/16/23	30 min	10	Review Processed Notes from Mtg 1 for completeness and accuracy All notes and kick-off information approved Questions Benefits of AWV
Call 2	12/7/23	30 min	10	Discussion of AWV benefits: What information is important to you? Which benefits mean something to you? What “sticks” or resonates with you? Homework assigned to write complete sentences about AWV benefits and any additional information, reactions, edits
Call 3	12/20/23	30 min	8	Community partners share homework assignment
Call 4	1/11/24	30 min	12	Target audiences discussed and finalized Distribution ideas (where, how) explored Materials characteristics (tone, look/feel, voice) explored and chosen
Meeting 2 (Virtual)	2/15/24	2.5 h	9	Recap progress Review and react to draft design mock-ups: elements we like/dislike (words, colors, images, “feel”) general designs selected and minor revisions requested
Meeting 3 (Virtual)	5/7/24	2 h	9	Update on other aspects of larger study Recap progress Review revised materials, identify final edits Review Distribution Guide for practices Celebration

Of the original 13 community-based patient participants at the first meeting, 11 continued with the project. One member stepped off due to an unexpected opportunity that demanded a substantial amount of time. Another member, while able to attend the first meeting in-person, did not have access to technologies needed for timely communication and participation and was unable to fully participate. If unable to attend a meeting or call, several members responded to emailed questions from the facilitation team to contribute their perspectives and opinions.

### BCT material dissemination

Materials developed by the BCT partnership were offered to practices participating in the larger NIH-funded studies. These included 8 community-based practices nested within 5 small organizations (ranging from 1 to 3 practice locations), 8 federally qualified health centers within 2 organizations, and additional practices belonging to 3 large health systems. confusion.

## Results

The Annual Wellness Visit BCT partnership identified key audiences for the materials they were developing, messages about the AWV that they felt were most important to share, the types of materials they believed would be most effective for the messages, and potential distribution strategies. Participants initially voiced some confusion over the concept of the AWV, the specific components required to meet AWV requirements, and the funding and logistical elements of the AWV. Participants had many questions about the medical jargon associated with billing codes, acute, chronic, and preventive care, and the history and physical exam components of the AWV. After the kickoff and ongoing conversations, participants were able to translate these concepts into patient relevant, actionable messages.

### Audiences

As expected, the BCT group deemed that the primary audience for AWV information was people aged 65 and older with Medicare (those who are eligible for an AWV), as this group comprises the largest percentage of Medicare patients. However, additional groups that might benefit from learning more about the AWV were primary care clinicians, clinical staff, and family members of those eligible for the AWV. The BCT group identified and discussed their personal experiences with lapses in knowledge in all of these groups.

### Messages and materials

A primary theme that emerged from the evidence-based AWV education presentation was the benefit of AWVs – benefit to patients, practices/clinicians/systems, patient-clinician dyads, and society. As shown in Table 4, a variety of benefits apply to each group, including potential prevention of illness or injury, potential for increased practice revenue, improved patient-clinician relationship, increased preventive care, and improved chronic disease control to name a few.

**Table 4:** Benefits described by participants for patients, providers, practices, and society.

Patients	Benefits to Providers/Practices/Society
More preventive care is completed People with AWVs might have fewer ER visits and hospitalizations Get to know your PCP better, and their team AWVs can help build the relationship between the patient and the clinician/practice Provide more time for addressing non-urgent issues that might improve health - My PCP knows my medical history best Early identification of potential problems, address them to prevent illness or injury Advance care planning is part of the AWV At our age, it's always great to get checked out medically Get healthy, stay healthy Asks questions that are outside a regular visit for a specific illness Whole person care Identify/detect health issues that might not otherwise be noticed	AWVs can generate more revenue AWVs may be a way to achieve higher quality care for the practice, increase revenue Can do other care at the same time as an AWV. And bill for that as well PCPs get to focus attention on factors that they might not otherwise address Important component of value-based care Benefits to Society Preventive care saves US/taxpayer money (People who get AWVs might have fewer ER visits/hospitalizations, prevention of disease) Other Benefits for Providers/Practices AWVs do not get in the way of other acute care visits for other patients Answer the question – “If I'm not sick, why do I need this?” If patients knew more about the AWV, they might not mind all the questions and forms Schedule my next AWV before I leave the office from my current AWV or other visit Team approach. Lots of AWV stuff can be done by other staff and primary care team

Over the course of several calls, the group identified several concepts related to the benefit of AWVs to translate for the targeted audiences. For patients and family members, this included preventive care, more time with their provider, building relationships with their provider, getting healthy and staying healthy, and awareness of AWVs. Sets of succinct “main messages” were crafted by the community group partners to address each concept. Examples of provocative messages included “Don't get left behind,” “What do I want to do this year?”, and “If you could prevent a health problem, would you?” A message aimed at family members asks the question, “Is someone important to you over 65 years old?” These main messages were used in a series comprised of twelve 11”x17” posters and twelve 4”x9” rack cards. Each poster had an open space for practices to add their own facility name, logo and contact information. Additional “detailed messages” provided more information about aspects and components of the AWV that the group deemed particularly important. Detailed messages were included on the backside of the smaller rack cards. See Table 5 for major themes and detailed messages within each theme.

**Table 5:** Final main themes and detailed messages included in materials.

General Concept	Main Messages (appear on poster series; select messages included on rack cards)	Detailed Messages (appear on backside of rack cards)
Prevention and preventive care	If you could prevent a health problem, would you? Schedule your AWV.a Tell your primary care team that you want your AWV.	Feeling fine? An AWV is still good for your health. A wide range of important health issues can be prevented. An AWV increases the number of preventive services you receive. A few examples include vaccinations, screenings, and mental and emotional health support.
Time, more time with doctor	Need more time? Get more time with your healthcare team. If you have Medicare, you get an AWV. Get more time to talk with your healthcare team. Schedule your AWV now!	An AWV gives you more time with your healthcare team to discuss you. AWVs are about caring for you as a whole person.
Relationship (relational not transactional)	How are you doing? I care about you. Your Medicare benefits include an AWV. Please schedule your AWV today. Make sure your primary care team knows what’s on your mind.	An AWV focuses on your needs. You and your healthcare team determine how best to use this time.
Getting healthy, staying healthy	How am I doing? What can I do to feel a little better? Do I need a little more time to talk with my doctor? Your AWV can promote your physical, mental, and emotional well-being. If you have Medicare, your AWV is easy, thorough, and important. Tell your doctor that you want your AWV!	A wide range of topics can be covered, such as vision, fall risk, pain, fatigue, depression, nutrition, medications, determining the care you want to receive should you be unable to communicate your wishes…to name a few! An AWV helps your healthcare team keep you healthy and identify areas that need attention.
Awareness of AWV (lack of)	Is someone important to you over 65 years old? YOU can help them GET and STAY healthy. Make sure they know about their AWV benefit from Medicare!	AWVs are not the same as an “annual check-up.” Many types of healthcare providers can do an AWV with you. Physicians, nurse practitioners, health educators, dieticians, and pharmacists. An AWV can include a physical exam – but an exam is not required. You and your healthcare team determine how best to use this time. Cost: Annual Wellness Visits are free to you. Your healthcare team may combine your AWV with a second visit type. You may get a routine charge or co-pay for the second part of the visit.
	Don’t get left behind. Join us. Do you have Medicare? Get more time with your doctor. Talk about what matters to you. Look at your calendar and schedule your AWV!	

a AWV, Annual Wellness Visit was written out in the printed materials.

b The target audience for this message was friends and family of people aged 65 and older. Other messages were written more directly to people aged 65 and older.

**Figure 2:**
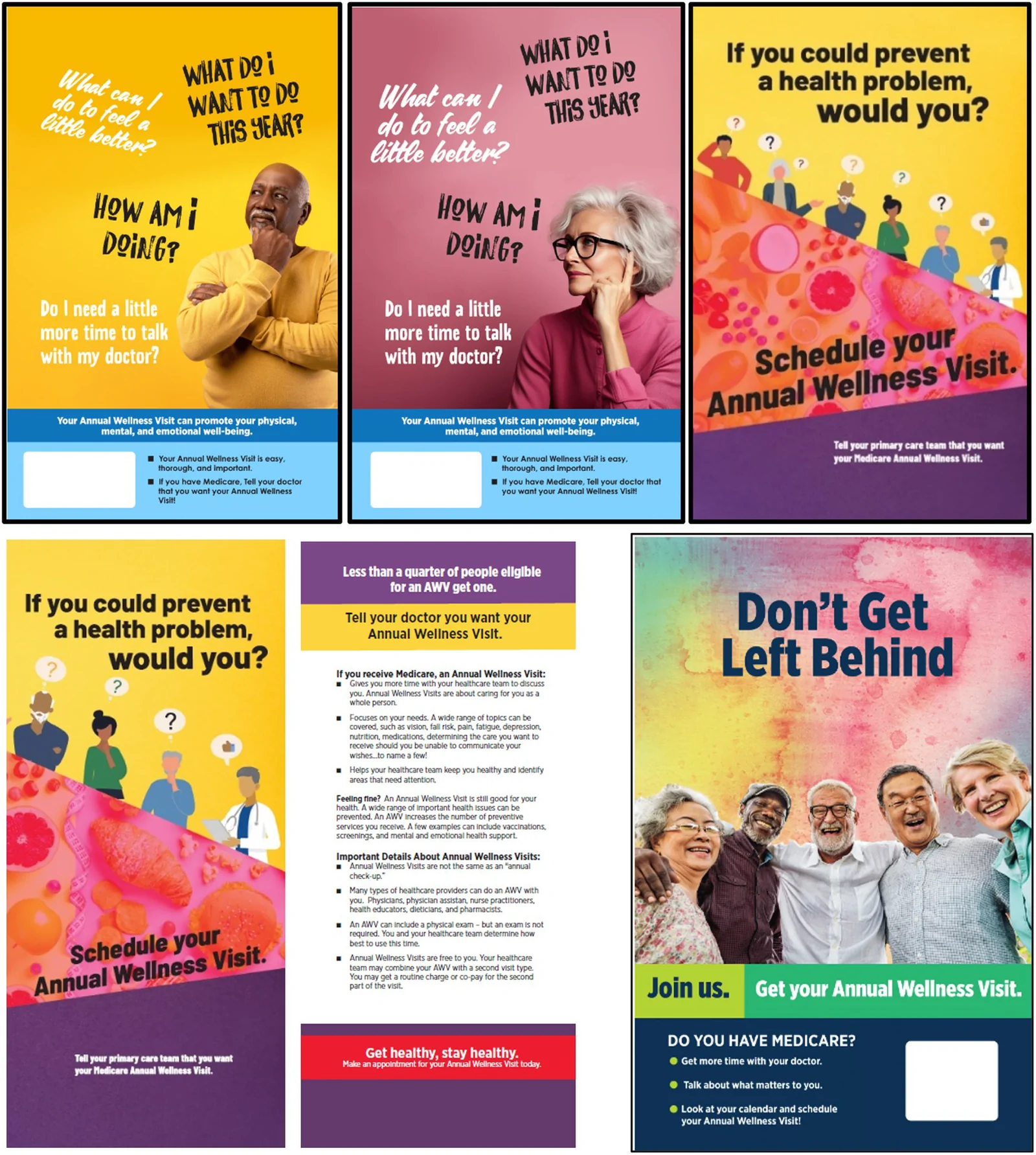
Annual wellness visit BCT messages and materials. Icons, illustrations of patients, and checklists reinforce the wellness message.

The group wanted these patient-facing materials to have the following characteristics: large print, clean fonts, colorful, positive information, one or two crucial sentences, not too polished but not cartoonish, and images of diverse people. In fact, images of several of the community partners were included in materials. Figure 2 provides several examples of materials.

The important messages were also collated and modified so that they could be included in electronic messaging from the clinic through emails or patient portal messages, newsletters, and social media. While the BCT group wanted to create a series of videos that could be offered to local television stations as public service announcements, the project budget and timeline did not allow for this more expensive product.

For practices, key concepts included revenue generation, higher quality care, care efficiency, team approach, and relationship with patients. The overarching main message was “Stronger Relationships, More Revenue, Better Health: the magic of the Annual Wellness Visit.” Detailed messages included statistics on the underutilization of AWVs; the benefits of more time with patients; higher quality care; the ease of a team-based approach to AWVs; and a four-step guide to scheduling and billing for AWVs. These messages were disseminated to practices through a Distribution Guide (36). This four-page guide, described further below, also included a message to practices from the BCT community participants emphasizing the importance of AWVs from their perspectives and supporting practices' efforts to include AWVs in their menu of services.

### Distribution strategies and uptake

The final products were offered free to practices participating in the larger NIH-funded studies. Given their wider appeal, digital images were also offered free of charge to non-study clinics wishing to use them. A website link for ordering materials and digital images was disseminated to the participating practices and through other collaborative efforts (37).

The Distribution Guide that was created for practices included suggestions for where to display materials in the practice setting. The group encouraged practice teams to be creative and hang the large posters all around the practice (e.g., waiting rooms, examination rooms, bathrooms) and place rack cards in multiple locations around the practice where they could be picked up by patients or handed out by clinicians and staff. In addition to practice settings, the group also wanted the opportunity for people to see these materials in other locations, such as pickleball courts, community facilities, senior centers, libraries, hair salons and barber shops, pharmacies, and churches and synagogues. This strategy could increase family members' and other community members' exposure to information about AWVs and, hopefully, help to initiate conversations with their loved ones over age 65 about getting an AWV.

Practices participating in the larger NIH-funded studies were given the opportunity to order materials for free. Six organizations representing 15 small community-based and federally qualified health center practices ordered 20 different types of items: 10 posters and 11 rack cards. In all, the practices ordered 905 items: 170 posters and 735 rack cards. The most popular images included messages about preventing a health problem, and one that used the phrase, “Don't get left behind. Join your friends, Get your AWV.” (Figure 2). Additionally, nine practices ordered digital images of the posters. Only two practices elected to include their logo on the materials. Providers and staff belonging to larger health organizations expressed interest in the materials but noted it would be onerous/lengthy/infeasible to obtain organizational approvals to display these types of materials in their practices.

## Discussion

This national BCT translated complex ideas and medical jargon around the AWV into messages and materials that were relevant to patients who might access their Medicare AWV benefit. These messages created by and for people aged 65 and older may be crucial to increasing patient knowledge and understanding about the existence and purpose of the AWVs, and have the potential to stimulate use of this underutilized Medicare benefit. BCT participants identified central messages regarding the AWV: benefits to patients of better health, benefits to providers and practices of increased revenue, and benefits to both parties of stronger provider-patient relationships.

A key message in the BCT materials focused on using the AWV to build stronger provider-patient relationships. Yet due to organizational and time constraints, many practices are moving toward incorporating pharmacists and nurses to complete these visits (7, 38), thus leading to missed opportunities for patients to further relationships with their primary care providers. Increasingly, AWVs are being offered by pharmacies and by nurses associated with insurance plans, further hindering the potential to enhance provider-patient relationships. While in any setting the AWV provides the potential for patients and clinicians to discuss preventive care and advance care planning, it is unknown whether patient completion of preventive services differs based on the type of provider and their relationship with the provider seen for this visit. Spending more time with a provider was another important BCT group message, yet access to primary care providers is worsening (39) and some providers have noted that it is difficult to do AWVs when patients have a lot of competing needs (5). Differing provider and patient expectations for the AWV also may make it difficult for providers to dedicate time to AWVs.

Results from the BCT group included messages targeting primary care providers and practices, eligible patients over age 65 with Medicare, and family members who might encourage their parents or siblings to get an AWV. Importantly, this project identified the role of not just the patients eligible for an AWV, but also that of family members. As studies have shown that almost 40% of patients are accompanied to visits by a family member (40), this group is important to include when developing messages to encourage healthcare visits.

This project demonstrated the success of a nation-wide BCT group. Participants in the AWV BCT were active throughout the entire 7-month time period. Attendance was strong despite multiple time zones and other personal and professional commitments. And several members asked to continue meeting or join other similar projects. While BCT is a recognized evidence-based method for patient and community engagement, this is the first BCT conducted outside of a local or regional context. Focused on a community of Medicare enrollees over age 65, this BCT is a model of an effective tool for national engagement for a broad population.

While participating practices were offered the opportunity to order materials, they were not required to use them, and this project did not verify use of the materials. The messages selected most by practices matched the priorities noted by the BCT group, and included messages and images that focused on using the AWV as a way to prevent health problems, to get more time with their doctor, to have an opportunity to talk about what is important to them, and not being left behind their peers who are having an annual wellness visit. One barrier to utilization of the materials was the approval process required by practices belonging to larger health systems for use of patient-facing educational or marketing materials. These requirements limited the capacity of individual practices to offer the products created in this BCT in a timely manner for this project. Smaller and independent practices, not faced with these requirements, were quick to order materials for use in their practices. Use of a professional graphic designer, while not required, is a strength of this BCT, but may pose a challenge for organizations that may not be able to afford a professional graphic designer. A professional graphic designer familiar with the BCT process of patient and community input offers the opportunity to create messages and materials with professional details and design elements in addition to more individualized topic content.

Further investigation is required to examine how different subgroups respond to the materials, particularly patients with lower rates of AWVs, such as those with lower income or from rural areas (2, 41). Additional investigation is also needed on how to best deliver messages about the AWV to older adults, whether by electronic health record patient portals as done in other studies (18, 19), or in the office as posters, flyers, or rack cards.

### Limitations

It is unknown whether use of the AWV materials actually stimulated patients to ask for or schedule AWVs, or whether these materials might have shifted the views of people who may not have recognized the importance of prevention. Future work is needed to more broadly evaluate the effect of the materials on patient knowledge about the existence and purpose of AWVs, on the likelihood of making an appointment for an AWV, and on patient attitudes about wellness and prevention. These materials were developed by a diverse group of patients from across the United States, suggesting that they may appeal to a broad range of patients. A snowball recruitment method may limit breadth of reach and diversity. However, recruitment began with a national organization (NAPCRG) which provided strong networking to a wide audience of potential participants.

## Conclusion

Boot Camp Translation successfully created messages and materials about the free Medicare AWV benefit that are meaningful and relevant to a diverse group of Medicare enrollees. There has been uptake by practices participating in an NIH-funded study seeking to increase AWVs. The materials created by this community and patient engaged group may offer primary care practices throughout the United States a resource to support their own efforts to increase patient engagement around the Medicare AWV.

## Data Availability

The raw data supporting the conclusions of this article will be made available by the authors, without undue reservation.
